# Role of Dilution Rate and Nutrient Availability in the Formation of Microbial Biofilms

**DOI:** 10.3389/fmicb.2019.00916

**Published:** 2019-04-30

**Authors:** Milos Legner, David R. McMillen, Dennis G. Cvitkovitch

**Affiliations:** ^1^Discipline of Microbiology, Faculty of Dentistry, University of Toronto, Toronto, ON, Canada; ^2^Department of Chemical and Physical Sciences and Impact Centre, University of Toronto Mississauga, Mississauga, ON, Canada

**Keywords:** biofilms, chemostat, mathematical models, dilution rate, nutrient availability

## Abstract

We revisited the mathematical model of the chemostat and examined consequences of considerably decreasing the concentration of limiting nutrient in the inflow for the growth of both the planktonic and biofilm cells in the chemostat tank (fermenter). The model predicts a substantially lower steady-state biomass of planktonic cells in response to decreasing inflowing nutrient concentration. Contrarily, the steady-state concentration of nutrient inside the fermenter is expected to remain the same, as long as the inflowing concentration does not fall below its value. This allows the biofilm cells to grow at a rate regulated only by the exchange rate of the medium (dilution rate). We maintained a strain of *Enterococcus faecalis* in a chemostat of our own design with limiting nutrient in the inflow set near saturation constant at three dilution rates (0.09, 0.28, and 0.81 h^-1^). The highest dilution rate was near the critical rate calculated by the model. The one-day total biofilm buildup was 21× larger and its estimated growth rate 2.4× higher at highest dilution rate than at the lowest one. This increased biofilm formation with increased dilution rates is in agreement with previously published data on pure and mixed continuous flow cultures.

## Introduction

A large amount of both experimental data and mathematical description of biofilm buildup has accumulated in the past (Rittmann and McCarthy, 1980; Evans et al., 1994; Beyenal and Lewandowski, 2002; Vinogradov et al., 2004; Wessel et al., 2013; Fernandez et al., 2017), yet little attention has been paid to the general validity of the chemostat theory in the context of biofilm formation. Originally, in the 1950–1960s, mathematical/engineering models of microbial growth dealt only with the suspended biomass, i.e., the planktonic cells. Researchers considered the “wall growth” to be an artifact whose presence perturbed or complicated experimental results, and did not view biofilms as a subject of analysis in themselves.

A chemostat as a physical instrument allows bacterial populations to grow at a constant rate for an indefinite period of time. As a mathematical model, the chemostat is simple, realistic, and thoroughly tested against the physical instrument by several generations of researchers (Bull, 2010).

Even though the chemostat model itself does not include a biofilm component, it calculates a “common currency” for both plankton and biofilm, i.e., concentration of substrate limiting the growth of the strain in question. As soon as a cell attaches to a submerged surface (underlying substratum material or previously attached cells), the current concentration of substrate ceases to be the limiting factor for its survival which creates a competitive advantage for attached cells over the cells still in suspension. The difference between the two types of cells is in prerequisites for surviving in the system: The suspended cells will be washed from the tank unless they divide at a rate dictated by its dilution rate. The biofilm cells can adjust to lower concentration of substrate by lowering their growth rate without punishment, their challenge, however, is to stay attached. Adhesion is a key trait that biofilm cells need to succeed in competition. It is accomplished using attachment factors and extracellular polymers. The production of extracellular matrix varies among strains and may result in eliminating the less producing cells from the systems by sloughing (Schluter et al., 2015).

The mathematical model of the chemostat devised by Novick and Szilard (1950) and corroborated by experimental evidence (Herbert et al., 1956) provided in essence a dynamic budget of materials in a well-mixed container with an input of the limiting nutrient (substrate) and output (outflow) of suspended bacterial biomass plus unused substrate. The current exploration deals foremost with allocating the resources between the suspended and attached component of a microbial population ruled by dilution rate and is based on the dynamic budget generated by the chemostat model. The experimental part of this study is rather a specific example (case study) that could be modified by changing its physical attributes, e.g., size and shape.

A part of the following analysis reiterates the structure of the theory of the chemostat and a reader not fully familiar with the topic is recommended to read more comprehensive texts such as by Kuenen and Johnson (2009), while the original ideas have long been established (Monod, 1942; Novick and Szilard, 1950).

The dynamic budget of the chemostat model is determined by three constants: the bacterial strain’s maximum growth rate (*μ*_max_), its saturation constant (*K*_s_), and a biomass yield coefficient (i.e., conversion efficiency, *Y*), all for a given limiting substrate.

For probing the applicability of the chemostat model to biofilm growth rates, we need to presume that the growth kinetics of both planktonic and biofilm cells of a given microbial species in relation to their nutrients (substrates) is similar, despite the differences in gene expression between the two phenotypes (Li et al., 2001; Rice et al., 2007). We will also assume that during population growth, the biomass of both planktonic cells and the cells within the top layer of biofilm, doubles at regular intervals, i.e., the growth is exponential. This allows us to calculate the exponent (specific growth rate, *μ*) from the increment of the natural logarithm of the biomass (*X*) plotted against the increment of time (t).

(1) $$X(t)=X_{0}e^{\mu t}$$

(2) $$\mu =\frac{\ln[X(t_{2})/X(t_{1})]}{t_{2}-t_{1}}$$

**FIGURE 1:**
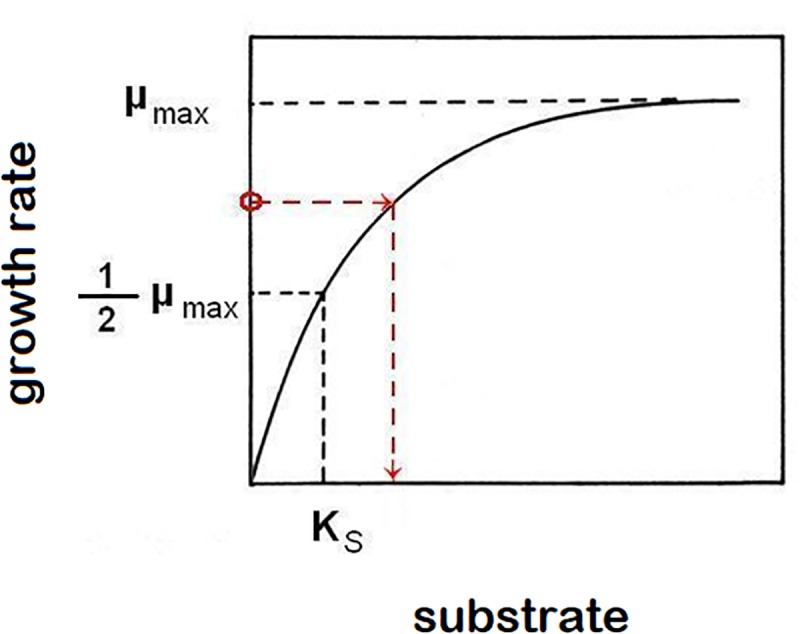
Monod’s function of saturation curve. The function asymptotically approaches highest growth rate value *μ*_max_. Saturation constant *K*_s_ is described as the limiting-nutrient (substrate) concentration that supports a half-maximum growth rate. In the chemostat, growth rate (μ) equals dilution rate (*D*). This causes a reverse effect, when the growth rate of cells in suspension controls the concentration of limiting nutrient (example is shown by red broken line, but any point of the function represents that relationship).

Equations (1) and (2) are sufficient to characterize growth in a closed system (static culture) with high initial nutrient concentrations to support it. In order to describe the growth of populations under changing nutrient conditions, we must use kinetic parameters to characterize the relationship between growth rates, and the availability of nutrients. Experimental data typically follow the hyperbolic Monod’s function [Monod, 1942; equation (3), Figure 1], a saturating function (identical in form to a first order Hill function or a Michaelis-Menten function) parameterized by two kinetic constants for any particular strain of microorganisms and any limiting nutrient (substrate, *S*): The maximum specific growth rate *μ*_max_ and the saturation constant *K*_s_ (defined as the nutrient concentration supporting growth at half the maximum rate). The growth rate at a given limiting nutrient concentration is approximated by the function:

(3) $$\mu (S)=\mu_{\max}\left(\frac{S}{K_{s}+S}\right)$$

For the planktonic biomass, *X*, in the chemostat, substrate-dependent growth μ(*S*) increases the biomass, while dilution at rate *D* (volumetric flow rate per volume of the container) decreases the biomass, leading to the rate equation:

(4) $$\frac{dX}{dt}=\mu (S)X-DX$$

When they reach a steady-state concentration, the cells in the chemostat will have adjusted their substrate-controlled growth rate so that it matches the dilution rate [*μ*(*S*) – *D* = 0; both values have the same physical dimension (h^-1^)]. The steady state value of the planktonic biomass, 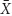, depends on how efficiently the substrate can be converted into cells, and this is typically characterized with a strain-specific empirical yield coefficient, *Y*: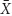 = Y (S_r_ 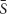), where 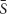 is the steady-state limiting substrate concentration and S_r_ is the in-flowing (“reservoir”) concentration of the limiting substrate.

As the planktonic cells adjust their growth to match the dilution rate, we can equivalently view this as the amount of residual substrate being a function of the dilution rate. Using equation (3) in μ(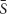) = D and solving for 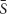, we find that:

(5) $$\bar{S}(D)=K_{s}\left(\frac{D}{\mu_{\max}-D}\right)$$

The control of a residual (steady-state) concentration of substrate in the chemostat by the link of growth rate to a hydraulic parameter *D* (dilution rate) shows that not only does the population’s specific growth rate depend on the concentration of a limiting substrate, but conversely the specific growth rate determines the concentration of substrate that cannot be further utilized (Figure 1).

The coupling between the planktonic cells and a growing biofilm comes through the level of free substrate that the planktonic cells leave available for the biofilm. The initial monolayer of attached cells, and the surface layer of cells in a growing biofilm, are in contact with the ambient fluid and are thus exposed to the steady-state substrate level, 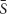 (*D*), determined by the planktonic biomass’s growth. Higher dilution rates lead to faster planktonic growth, but also to higher substrate availability: the rapid dilution provides a large supply of incoming substrate, and the fast-growing planktonic cells are not able to use it all up before being washed away in the outflow. This leads to a prediction: more rapid dilution rates in a chemostat should support higher rates of biofilm growth, because the biofilm’s surface will be exposed to higher substrate concentrations.

Consider the case of planktonic cells growing at half their maximum growth rate. When the inflowing concentration of the nutrient is much higher than *K*_s_ and the dilution rate (*D*) is kept equivalent to half-maximum growth rate in a well-mixed container, the planktonic cells would quickly establish a steady state population that would maintain the residual concentration of the nutrient at *K*_s_. This translates in the biofilm growing at half-maximum rate. If the hydraulic conditions are kept the same, but the concentration of nutrient in the fluid entering the system would drop to *K*_s_, the planktonic component of cell population would substantially diminish, but the surface biofilm cells would receive the same input of the nutrient as before, and would grow at the same half-maximum rate. Decreasing the dilution rate in this situation would favor planktonic cells by making the substrate more available to them and less available to the biofilm. Increasing the dilution rate without eliminating the plankton biomass would only be possible if we increase the concentration of nutrient in the inflow.

While this prediction follows from the original Novick and Szilard (1950) model, the mathematical description does not explicitly include any representation of biofilm. On the other hand, along the same lines as the experiment of Herbert et al. (1956), which validated the chemostat theory using a single bacterial strain, it seemed sensible to perform an empirical test of biofilm formation at three substantially different dilution rates in a chemostat culture of *Enterococcus faecalis* and compare it with predicted theoretical concentrations of limiting nutrient.

It should be noted that the above considerations apply only to biofilms supported by nutrients dissolved in the ambient liquid. Biofilm formation on wetted surfaces of the soft tissues of multicellular organisms (such as human respiratory or digestive tract) that provide nutrients to bacteria by themselves is more complex and obviously driven by additional mechanisms (Tart and Wozniak, 2008; Schluter et al., 2015).

## Materials and Methods

### Static (Batch) Culture

Initial exponential growth rates of *E. faecalis* (ATCC 29212) were estimated in a dilution series of Trypticase Soy Broth (TSB). The initial concentration of the medium (Pancreatic Digest of Casein 17.0 g L^-1^; Soy Peptone 3.0 g L^-1^; NaCl 5.0 g L^-1^) was divided in half at each consecutive dilution while the concentration of 28 mM glucose and 66 mM phosphate buffer (Sorensen at pH = 7.0) was kept constant in all dilutions of the TSB. The specific growth rates were calculated from the increments of optical density (OD_600_) readings at 30-min intervals after inoculating an overnight culture in the dilution series at 37°C.

### Laboratory Model

#### Strain and Medium

The same strain of *E. faecalis* (ATCC 29212) that served for estimating the initial exponential growth rates was used in the laboratory chemostat experiments. A single dilution of TSB was used in all runs, i.e., 1/16 (6.25%) with 28 mM glucose, and 66 mM phosphate buffer (Sorensen at pH = 7.0).

#### Chemostat

**FIGURE 2:**
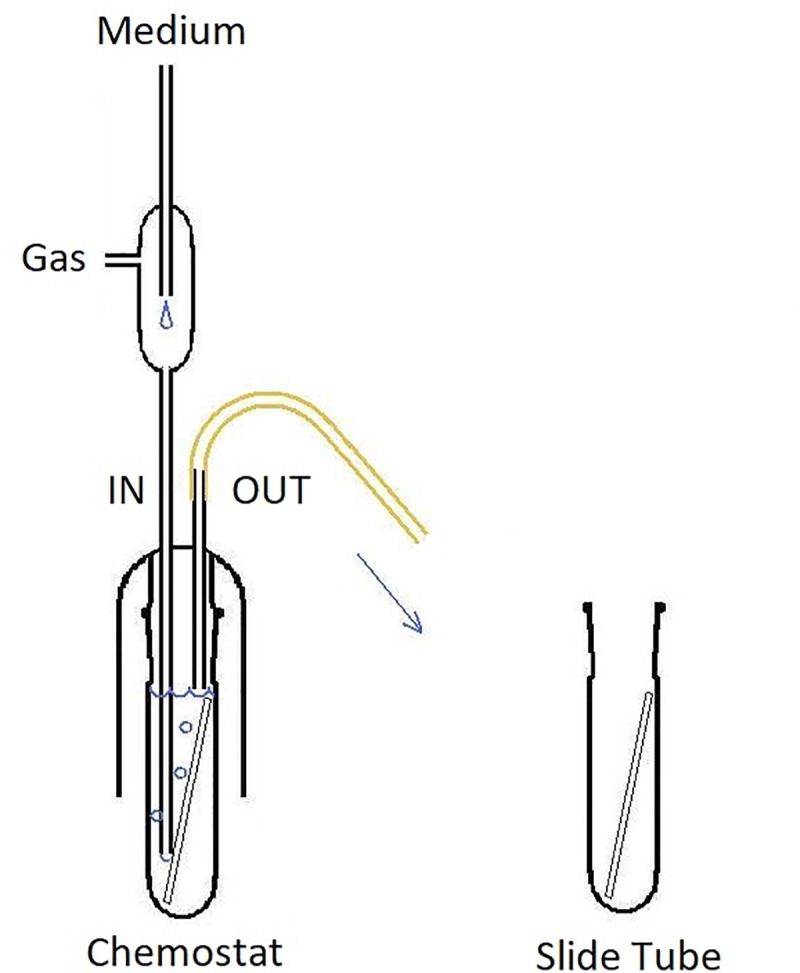
Design of the glass chemostat tank. IN, inflow tubing reaching close the bottom of fermenter; note the flow interrupter assembly mixing sterile medium that drips through the space protected with HEPA-filtered gas flow. OUT, overflow tubing (keeping a constant fluid volume of the chemostat). Slide Tube, container allowing aseptic exchange of the chemostat contents including the biofilm slide.

We used a simple all-glass tank of our own design that allowed aseptic exchange of the chemostat contents including the biofilm slide (Figure 2). Volume of the tank was 57 mL, the inner surface including the surface of biofilm slide (40 cm^2^) was 184 cm^2^. The medium was aseptically metered using a peristaltic pump (Econo Gradient Pump 731–9001 Bio-Rad Laboratories, Ltd.) into tubing that also provided exchange of gases and mixing of the chemostat contents. The sterile gas flow (3% CO_2_ in air) secured dispersion of each medium drop as its volume exceeded medium flow multiple times. The tank itself consisted of a glass shield holding inflow and overflow tubing, and an exchangeable slide tube carrying a biofilm slide amenable to CLSM imaging. The surface of the slide not exposed to gas bubbling was used for treatment with fluorescent probes and viewing. The continuous flow system was kept in a constant-temperature (37°C) room. Originally, 5 mL of an overnight culture was aseptically transferred to the chemostat apparatus in a sterile slide tube. The pump was activated and run until the culture vessel was full and then stopped. Flow resumed after a 2-h rest and was kept at a nominal flow rate. Depending on the current run, it was set to maintain the dilution rate *D* at 0.81, 0.28, and 0.09 h^-1^, respectively (in that order). After a prolonged growth of the culture at a constant *D*, a biofilm slide was exposed to it for 24 h.

#### Handling of the Biofilm Slide

The biofilm slides were cleaned with Tergazyme, rinsed with RO water and wiped with sterile Kimwipes, then they were inserted in wrapped slide tubes, and autoclaved. No coating of the slides was performed. For starting the slide exposure, the pump was stopped and the slide tube with culture was aseptically exchanged for a sterile empty tube. In a biosafety cabinet, a sterile slide was inserted in the culture. Then the slide tube was returned to the chemostat apparatus and the flow resumed. After 24 h of exposure, the pump was stopped. The slide tube containing the biofilm slide was aseptically exchanged for a sterile empty tube. In a biosafety cabinet, the slide was removed to a sterile petri dish and the tube with culture was returned to the chemostat apparatus. The slide was then overlaid with BacLight Live/Dead (Invitrogen, United States) probe solution [mixture of 1.5 μL Syto 9 and 1.5 μL of propidium iodide (PI) from the bacterial viability kit in 1 mL PBS] and after 15 min carefully rinsed with PBS for the same period in another petri dish. The surface treated with the fluorescent probe was then covered with a coverslip and the edges sealed with nail polish to prevent evaporation. The bottom side of the slide was cleaned with 95% ethanol.

**FIGURE 3:**
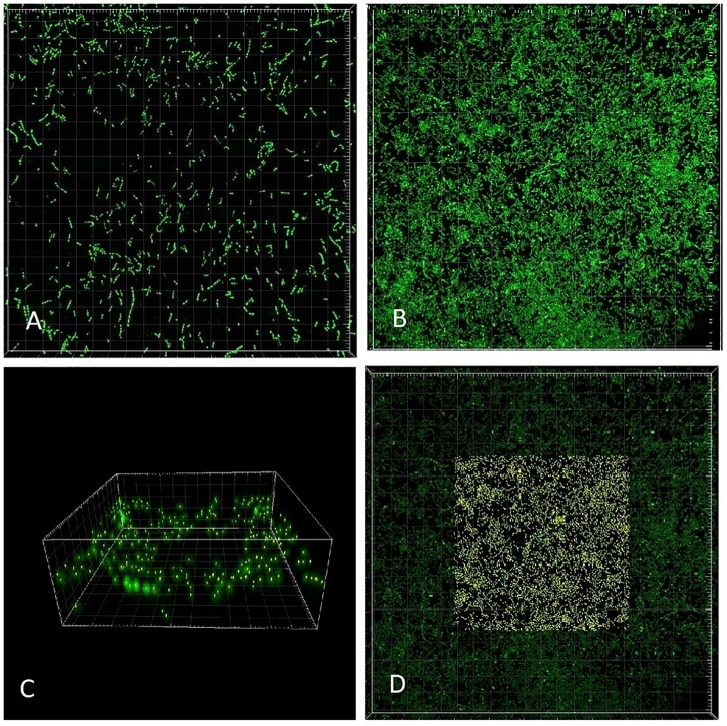
CLSM (Syto 9) 3-dimensional rendering of *Enterococcus faecalis* biofilms at two different dilution rates (*D*). **(A)** *D* = 0.09 h^-1^ and **(B–D)** *D* = 0.81 h^-1^. Cells marked with Imaris Point Viewer **(C)** entire image and **(D)** central 25% of image volume (same CLSM file as **B**). Side of the square base of image **(A**,**B**,**D)** 203.2 μm and side of the square base of image **(C)** 25.4 μm.

The slide was transferred to a confocal laser scanning microscope (CLSM; Zeiss LSM700) and processed promptly. Scanning was performed in inverted position (coverslip at the bottom) with a 63× oil objective. Green fluorescence of Syto 9 was excited with a laser wavelength of 488 nm (Figure 3), while the red fluorescence of PI representing non-viable (membrane-compromised) cells was excited with the 555 nm line. The number of cells captured in Syto 9 stacks was considered a total cell count. Double-labeled cells (emitting both green and red fluorescence) were regarded as non-viable (cf. Klug et al., 2016).

### Image Analysis

Images of individual bacteria acquired with CLSM using DNA probes are often poorly defined for the purposes of quantification with “classical” image analysis, as the cell size nears the resolution limits of optical microscopy including CLSM (Centonze and Pawley, 1995). Growing biofilms present additional challenges while transforming themselves from monolayer to multilayer (3-dimensional) structures. Post-acquisition computational analysis of optical microscopy images has advanced significantly in recent decades (Thomann et al., 2002). For purposes of the analysis of near sub-resolution objects, local maximum has been defined as a voxel (3-D picture element) that can be used as a seed point for a segmentation of objects (Gniadek and Warren, 2007). We have utilized the capability of proprietary software Imaris (version 7.6.4, Bitplane United States, Concord, MA, United States) to detect local maxima in 3-D CLSM stacks. Imaris Surpass Module was used to locate and enumerate individual cells on the surface of biofilm slides (Figure 3C,D). The module is interactive and allows modifications of algorithm according to specifics of a data set. Because individual CLSM images inherently differ in quality, local maxima quantification can be visualized by the user to confirm that the marking resolves the individual cells with sufficient accuracy. In CLSM images of high local maxima density, the algorithm allows to quantify a part of the file (region of interest; Figure 3D) before finalizing the count.

## Results

### Kinetic Parameters of Bacteria

**FIGURE 4:**
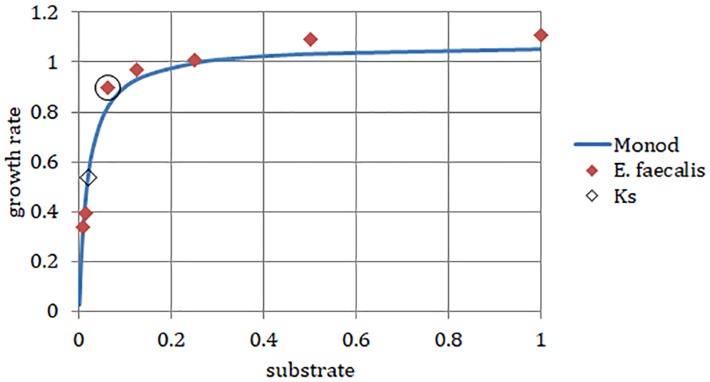
*Enterococcus faecalis* static cultures. Data points, highest growth rates attained at respective concentrations of TSB (encircled point: concentration used in the laboratory chemostat). Monod, hyperbolic function curve using parameters calculated from data points (*μ*_max_ = 1.07 h^-1^; *K***_s_** = 0.019). Horizontal axis, concentration of TSB (arbitrary units reflect a fraction of the full concentration from no dilution to 1/128, omitting the dilution 1/32). Vertical axis, growth rate (h^-1^).

We chose a strain of *E. faecalis* (ATCC 29212) for estimating initial rates of exponential growth at serial dilutions of its common culture medium, Trypticase Soy Broth (TSB). The initial concentrations of the medium were divided in half in each consecutive dilution. When we plotted the highest growth rate reached in the respective batch culture against dilutions of the original complex medium, the graph looked like the classical Monod’s saturation curve. Even though the original function had been designed for a single limiting substrate, it allowed estimating the concentration of sterile medium just sufficient to support population of bacteria growing at a given rate (Figure 4). The parameters *μ*_max_ and *K*_s_ were calculated from a double reciprocal plot of *μ* and *S* (dilution of TSB) values. This way of estimating the above parameters is not regarded as the most accurate (Kuenen and Johnson, 2009), yet it was sufficient for examining the theoretical behavior of the *E. faecalis* culture.

### Mathematical Model of the Chemostat

**FIGURE 5:**
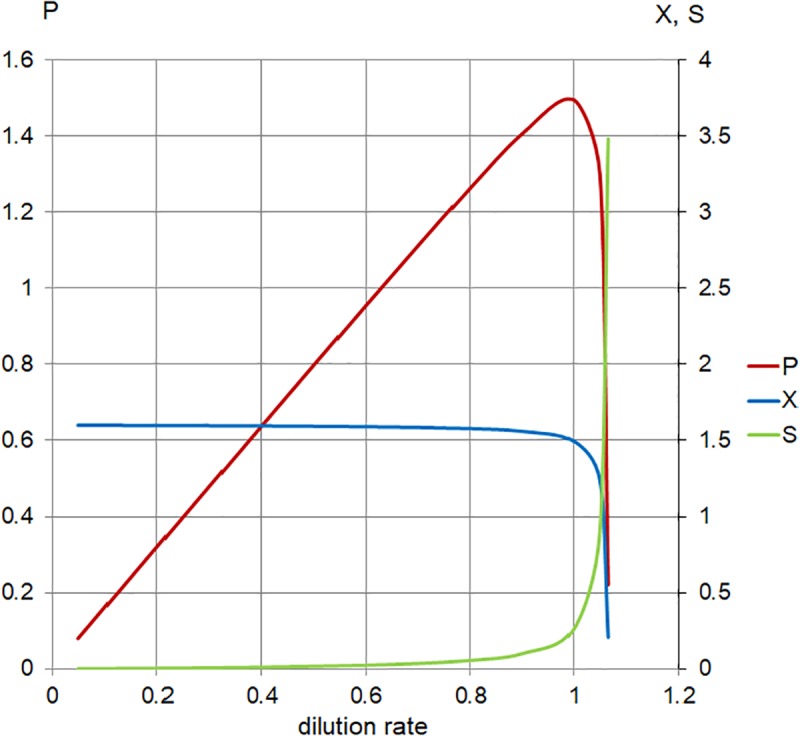
Calculated steady-state values for the model set in Table 1, Case 1. Concentration of limiting nutrient (*S*, green), suspended bacterial biomass (*X*, blue), and biomass production (*P*, red); left axis, *P* (arbitrary units/hour); right axis, *X*, *S* (arbitrary units); and horizontal axis, dilution rate (*D*; h^-1^).

We exploited the potential of the Novick and Szilard (1950) model of chemostat kinetics as a predictor of the steady-state values for the limiting-substrate concentration, 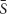 (*D*). To apply the model, we used two constants, *μ*_max_ (1.07 h^-1^) and *K*_s_ (0.019) derived from our *E. faecalis* static cultures. The third constant, the yield coefficient, was set as 40%, *Y* = 0.4 based on typical literature data for bacteria. Lastly, the fourth parameter (the concentration of limiting substrate in the inflow, *S_r_* ) had to be dramatically adjusted upward (we set it arbitrarily 4× higher than the full strength of TSB medium, i.e., 211× higher than *K*_s_) so that the model did not produce negative values at high settings of dilution rate, *D* (Figure 5 and Table 1). The model generated explicit steady-state values of substrate 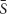 (*D*), suspended cell biomass [planktonic, 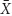
(*D*)], and its production (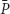 = 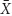 ⋅ D) covering the range of dilution rates equivalent to 0 – *μ*_max_. The arbitrary units of substrate and biomass could be easily converted to physical units, such as dry weight, carbon, etc.

**Table 1:** Kinetics of the chemostat model based on *Enterococcus faecalis* data.

Constants *μ*_max_ = 1.07 (h^-1^) *K_s_* = 0.019 (arbitrary units) *Y* = 0.4 (dimensionless)		

**Controlled parameters**		

	***S*_r_ (arbitrary units)**	***D* (h^-1^)**
Case 1	4.0	0–*μ*_max_
Case 2	0.06	0–0.8
**Computed variables**		
Steady-state substrate *(S)* (arbitrary units)		*S = K_s_ (D/μ_max_-D)*
Plankton biomass *(X)* (arbitrary units)		*X = Y(S_r_-S)*
Biomass production (*P*)		*P = D∗X*

*Arbitrary unit defined as the full concentration of TSB. Case 1, limiting substrate concentration in the inflow (S_r_) 4× larger than full TSB; Case 2, S_r_ 6% of full TSB). D (h^-1^), dilution rate.*

As the plot (Figure 5) indicates, increasing the dilution rate in the chemostat has the following effects: the steady-state planktonic biomass decreases, until it eventually drops to zero as the dilution rate rises to μ_max_ and the planktonic cells are no longer able to grow quickly enough to compensate for the dilution rate; the level of available substrate rises, until it eventually reaches a physically constrained maximum of S_r_; and the production of planktonic biomass increases to a maximum where the effects of increased dilution rate and decreased biomass are optimized, before falling as the planktonic biomass begins to drop.

**FIGURE 6:**
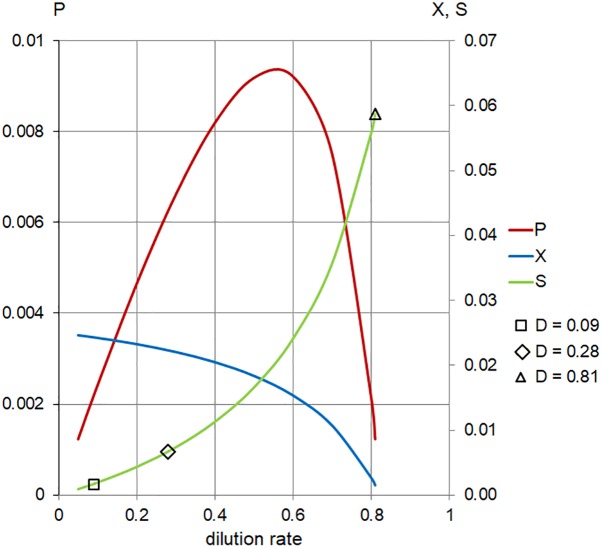
Calculated steady-state values for the model set in Table 1, Case 2. Concentration of limiting nutrient (*S*, green), suspended bacterial biomass (*X*, blue), and biomass production (*P*, red); left axis, *P* (arbitrary units/hour); right axis, *X*, *S* (arbitrary units); and horizontal axis, dilution rate (*D*; h^-1^). Markers indicate values of *S* for dilution rates (*D;* h^-1^) used in the chemostat experiments.

In order to address the effect of low (near *K*_s_ ) concentration of substrates in the inflow (*S_r_* ), we applied the same model at 1/16 of the full TSB medium (*S_r_* = 0.06). These conditions generated positive values of cell biomass in planktonic cells, 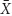
(*D*), only for

the range of dilution rates 0 < *D* < 0.8 (Figure 6 and Table 1). The outcome illustrates the core of the Novick and Szilard’s model, predicting that the concentration of limiting substrate is controlled by the growth rate of cells in suspension, which in turn equals the chemostat’s dilution rate. In this second plot of limiting nutrient, we used different scales on the vertical axes to show detailed changes in the low concentration of 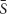 (*D*) accompanying extremely low values of 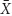 (*D*) and 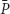, when the dilution rate (*D*) approached the highest value (0.8 h^-1^). However, comparing the same scale of 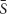 (*D*) concentrations in both graphs shows that that the calculated values were identical. In Table 2, theoretical steady-state substrate concentrations are displayed for three values of the dilution rate *D* that were implemented in the subsequent *E. faecalis* chemostat experiments. Corresponding values are marked in the graph on the limiting nutrient 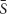 (*D*) – green line (Figure 6).

**Table 2:** Steady-state substrate concentration (*S*, arbitrary units) derived from the Novick-Szilard model (arbitrary unit = concentration of the original TSB medium).

Dilution rate *D* (h^-1^)	0.09	0.28	0.81
Substrate concentration at steady state (*S* )	0.0017	0.0067	0.0587
Multiple of *Ks* Value	0.09	0.35	3.1

*The same values were obtained for S_r_ = 4.0 and 0.06, when setting μ (h^-1^; growth rate) = D (h^-1^; dilution rate), as well as directly from the Monod’s saturation function.*

### Laboratory Model

We used a glass tank that allowed aseptic handling of the chemostat contents including the biofilm slide (Figure 2). The continuous flow system was inoculated with the same strain of *E. faecalis* as the static culture and fed with a single dilution of the medium (1/16 TSB). Three different dilution rates (*D*) were used to establish steady-state cultures: 0.09, 0.28, and 0.81 h^-1^. In each run, a sterile biofilm slide was immersed in the chemostat culture for 24 h. Before the slide was inserted in the culture, the flow was kept constant for a period of at least 9 residence times (*R* = 1/*D;* Table 3) since the chemostat culture had been transferred to a new slide tube. After one-day exposure, the biofilms were treated with fluorescent DNA probes. CLSM stacks capturing *E. faecalis* cells were taken and quantified using 3-dimensional image analysis.

**Table 3:** Parameters (P), experimental (E), and theoretical (T) values for the chemostat culture of *E. faecalis*.

Dilution rate (*D* ) (h^-1^)	0.09	0.28	0.81	P
Doubling time (h)	7.7	2.5	0.9	T
Percentage of *μ*_max_	8.4%	26.1%	75.6	P
Number of CLSM stacks	7	7	4	E
*R* of steady state	9	40	35	E
Cells cm^-2^	3.78 E+06	5.54 E+07	7.94 E+07	E
Initial density cm^-2^	4.36 E+05	N/A	N/A	T
Total biofilm *μ* (h^-1^)	0.09	0.20	0.22	T
Non-viable cells	72.2%	0.8%	4.3%	E
Percentage of highest *P*	35%	100%	20%	T

**FIGURE 7:**
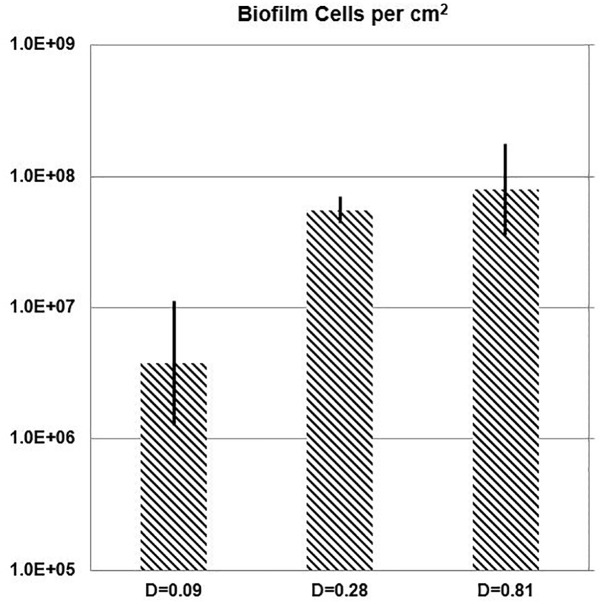
One-day biofilm. Cell density of *E. faecalis* on a biofilm slide glass surface (Syto 9) estimated with Imaris Point Viewer (bars indicate ± SD) at three different dilution rates (*D;* h^-1^).

While the chemostat culture was fed with a constant concentration of medium demonstrated to limit their growth rate, the quantity of *E. faecalis* cells in biofilm increased with the dilution rate. Over the range of tested dilution rates, *E. faecalis* built a 21-fold denser biofilm in response to a ninefold increase in the ambient dilution rate (Figure 7 and Table 3).

## Discussion

The chemostat theory of continuous cultures has been particularly helpful in industry, but not fully adopted in the fields of ecology and biomedical engineering. As early experimenters discussed (Herbert et al., 1956), some researchers rejected it, failed to understand it, or found it counter-intuitive. Similarly, an advantage of biofilms over suspended population regulated by the dilution rate may appear paradoxical. However, our example of the near-saturation-constant concentrations of inflowing substrate may help elucidate the kinetics behind this phenomenon.

### Laboratory Chemostat

In order to obtain reliable empirical data, we optimized our protocol for aseptic handling and accuracy. Shielding a glass-to-glass connection allowed contamination-free replacing entire fermenter (slide tube) for inoculation and sampling of biofilms. All additional operations were performed in a sterile biosafety cabinet. Back-flow contamination was prevented by a protective stream of sterile gas. The constancy and homogeneity of nutrient supply to the fermenter was improved by instant dispersion of medium by gas bubbles. A precision pump allowed control of a precise and stable dilution rate. Lastly, cell quantification was carried out by the combination of CLSM and 3-dimensional image analysis allowing adjustment of the algorithm to image condition.

In the rectangular-hyperbolic plot of Monod’s function, the *S_r_* value used in the chemostat culture lies near the junction of the two branches of the curve. Therefore, the steady-state substrate values [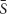 (*D*) ≤*S_r_*] were located on a steep part of the function, where small changes in nutrient concentration (*S*) seem to be correlated with large changes of specific growth rate (μ) and dilution rate (*D*; Figure 4). However, that perception is a result of using a linear scale in the Monod’s plot. In actuality, the calculated values of steady-state nutrient concentration increased 35 times in response to ninefold change of dilution rate between *D* = 0.09 and 0.81 h^-1^ (Table 2). This helps us understand the profound effect of translating dilution rate into biofilm buildup.

**FIGURE 8:**
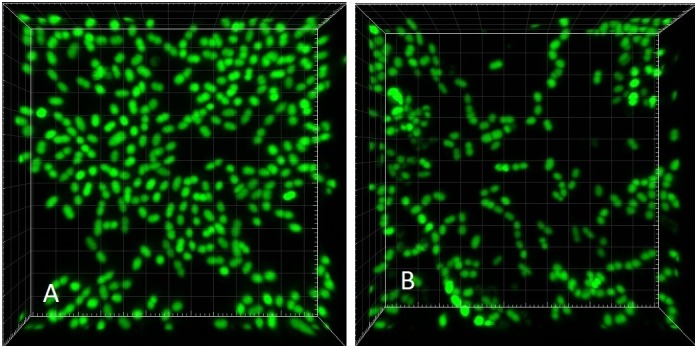
CLSM (Syto 9) 3-dimensional rendering of *E. faecalis* biofilms at two different dilution rates (*D*). **(A)** *D* = 0.28 h^-1^ and **(B)** *D* = 0.81 h^-1^ (same CLSM file as Figure 3C). Side of the square base of image 25.4 μm.

Visually, the biofilm at *D* = 0.09 h^-1^ was a “loose monolayer” (Figure 3A), at *D* = 0.28 h^-1^ appeared as “crowded monolayer” (Figure 8A), whereas that at *D* = 0.81 h^-1^ had a “loose multilayer” appearance (Figure 3C, 8B). It is to be expected that in multi-layer biofilms, the diffusion of nutrients through extracellular matrix to deeper layers is decreased resulting in a slower growth rate of deeper lying cells as well as the overall growth rate of the biofilm. The layered structure of biofilm made the effect of residual substrate concentration in ambient medium less obvious. On the other hand, being aware of the importance of extracellular matrix for the biofilm adhesion we had to assure its sufficient generation. Since exopolysaccharide is an essential component of matrix and its production depends on a saccharide source in the medium, we adjusted glucose concentration to 28 mM that proved to be a sufficient concentration not limiting the biofilm formation in pilot experiments. We kept this concentration constant in static cultures while diluting TSB medium for estimating initial rates of exponential growth of *E. faecalis*. This way the composition of the medium chosen for chemostat culture was identical with the dilution 1/16 of the medium for static cultures. In independent experiments, we were able to visualize extracellular polysaccharide and eDNA in the matrix. However, the matrix is not visible in CLSM stacks of this study where only the chromosomal DNA of bacteria is targeted by fluorescence probes.

As opposed to the assay with the middle and highest dilution rate, in the run at *D* = 0.09 h^-1^ the biofilm images showed cells dispersed on the surface of the glass, never forming a confluent layer, hence the matrix was expected to be minimal (Figure 3A). We presumed that before reaching this “final” density during 24 h of its development, the biofilm was exposed to ambient concentration of limiting substrate, same as the plankton cells and, as a result, grew at the rate dictated by the chemostat environment (equal to *D* = 0.09 h^-1^). Thus we re-calculated the “starting” number of cells per cm^2^ from the one-day estimates for the assay reversing equation (2) (Table 3). We can interpret this value as the number of planktonic bacteria that, during the 24-h period, have impinged on the surface of the slide and have been able to attach (thus the term “initial” is somewhat inaccurate). It is plausible to expect that these cells would continue multiplying at the rate determined by the ambient concentration of limiting substrate. Since this initial density calculated for *D* = 0.09 h^-1^ was its only good approximation, we used the value for estimating the growth rate of the biofilms in the remaining two assays (Table 3). Based on this calculation, the total growth of biofilm in the two remaining assays (*D* = 0.28 and 0.81 h^-1^) was much slower than the growth rate of plankton biomass (0.20 and 0.22 h^-1^, respectively).

A theoretical variable, the product of plankton biomass and dilution rate called production (P), represents the biomass that can be “harvested” (e.g., by capturing outflow of the chemostat), its physical unit being g L^-1^h^-1^ (Kuenen and Johnson, 2009; using the term “productivity”). It is not clear how to interpret this parameter in relation to biofilms, i.e., how it influences the attachment of planktonic cells to the submerged surfaces. If for instance, cell attachment would be proportional to production, then at higher P, biofilms would probably contain lower numbers of dead (dying) cells, since their frequency in steady-state (plankton) populations tends to be zero (Powell, 1956). Using these criteria, we can see that the run with *D* = 0.81 h^-1^ had more than a 5× higher content of non-viable cells than the run at *D* = 0.28 h^-1^, while the biofilm at *D* = 0.09 h^-1^ contained over 70% of these cells. At the same time, the mathematical model predicted the highest planktonic production at *D* = 0.28 h^-1^, which was more than 5× higher than at *D* = 0.81 h^-1^ (Table 3). The middle dilution rate assay (D = 0.28 h^-1^) with highest P had the lowest proportion of dead cells (0.8%). Therefore, the noticeable lack of significant difference between cell density at *D* = 0.28 h^-1^ and 0.81 h^-1^ could also be caused by a higher rate of attachment at *D* = 0.28 h^-1^ due to a higher production of plankton. It has been previously demonstrated (Sternberg et al., 1999) that the biofilm growth rate represents an average of a wider range of values. This biofilm heterogeneity is in a sharp contrast with the planktonic component of chemostat populations where the growth rate of most cells is expected to be close to the dilution rate (Powell, 1956).

Apparently, the tools used in this study allowed only a rough comparison between the mathematical and physical model. Even over standardized time, the biofilm biomass increase was not a direct measure of its growth rate or production. On the other hand, we have not yet fully utilized the versatility of our chemostat design. If employed in multiple tanks, it would enable researchers to dissect the plankton/biofilm interplay owing to its capability to transfer independently the biofilm-covered slide and/or the entire tank contents (plankton) to a new, sterile tank in real time. It would allow us using markers (e.g., constitutive fluorescent proteins; Ai et al., 2006; Shcherbo et al., 2007) to clearly distinguish between cells originating alternatively from neighbor biofilm cells or plankton cell population. The rates of attachment or biofilm sloughing could thus be quantified.

In order to corroborate the assumption that the newly attached bacteria would continue multiplying at the rate determined by the dilution rate before the biofilm becomes multilayered, it would also be possible to expose the slide to chemostat culture for shorter intervals in the second (*D* = 0.28 h^-1^, e.g., 8 h) and third assay (*D* = 0.81 h^-1^, e.g., 2.5 h). As the exposure would be shortened roughly in proportion to the plankton population doubling time (ln 2/*μ*; Table 3), the biofilm cell counts in these two assays would be presumably similar to those in the present study at *D* = 0.09 h^-1^.

In spite of all the above discussed issues, there was a clearly increasing trend of biofilm buildup between the runs at *D* = 0.09 h^-1^ and 0.81 h^-1^. Within the limits of dilution rates tested in this study, a substantially lower buildup occurred at a low dilution rate (*D* = 0.09 h^-1^; 8.4% of *μ*_max_) than at faster dilution rates (0.28 h^-1^ and 0.81 h^-1^; greater than 26% of *μ*_max_). *E. faecalis* developed a 21-fold denser biofilm in response to a ninefold increase in the chemostat dilution rate (Table 3).

### Dilution Rate and Nutrients

We established the *μ*_max_ and *K*_s_ constants for *E. faecalis* (ATCC 29212) after evaluating a series of batch cultures and applying the function of the saturation curve (Monod, 1942). Then, we were able to calculate a theoretical limiting nutrient concentration at the steady state (*S*). According to the mathematical chemostat model (Monod, 1950; Novick and Szilard, 1950), this variable is not sensitive to the concentration of the inflowing medium (*S_r_* ). However, there is a restriction of the *S* value by the fact that *S* ≤*S_r_* at all times. We used this rule for setting the limits for calculation of variables in continuous culture fed by medium with limiting nutrient concentration. Using the Monod’s function, we estimated the highest (critical) dilution rate still allowing a steady-state planktonic growth.

We fed the system with a concentration of nutrients supporting a growth rate lower than *μ*_max_, hence, the equations held only for a range of dilution rates (*D*) lower than its critical value. In our physical continuous culture, there was a trend for an increased rate of biofilm formation over that range. Since the calculated steady-state substrate concentration (*S*) was the only variable that increased with *D* at rates approaching its critical value, the rate of biofilm formation may be predominantly driven by the growth rate of attached cells due to increased ambient substrate concentration (*S*). Thus, the biofilm formation is controlled by the dilution rate (*D*) through the growth rate (*μ*) of the planktonic component. Higher planktonic growth rates resulted in increased biofilm growth while the planktonic biomass decreases.

The chemostat model with the parameters used for our *E. faecalis* cultures predicted the maximum planktonic biomass production when *D* approached the critical value (either due to availability of substrate in our “nutrient restricted” chemostat or approaching limits of the strain’s capacity for growth – *μ*_max_). This could also provide insight into the contribution of the planktonic biomass to the biofilm buildup. The calculated “production” variable (Figure 5, 6) is an amount of biomass grown in the fermenter but washed out by the passing nutrient medium. It may be proportional to the rate of attachment of planktonic cells and aggregates, i.e., colonizing the surface layer by outside biomass.

The steady-state model calculates the value of *S* regardless of the values of *S_r_* or *X*, i.e., solely on the basis of prevailing dilution rate *D*. As demonstrated in Table 2, this actually implies that for mere assessment of a steady-state substrate concentration, the Monod (1942) saturation function is sufficient. We can apply the Monod’s function to establish a more general definition of the effect of fluid exchange on biofilm formation. The highest *μ*_max_ values of microorganisms published thus far can be used for assessing the upper limit of *D* expected to influence the buildup. A good approximation could be 4.2 h^-1^, the highest value reported for a marine bacterium *Vibrio natriegens* (Eagon, 1962), which is considered the fastest growing prokaryote (Maida et al., 1973; Hoffart et al., 2017). Likely, a faster exchange of fluid than the corresponding dilution rate cannot further enhance biofilm growth.

### Shear Stress

During the last two decades, flow cells became a favorite tool for biofilm research (Heydorn et al., 2000; Foster and Kolenbrander, 2004; Benoit et al., 2010; Nance et al., 2013). These research or industrial flow-through devices harbor biofilms that appear to exist at fluid exchange rates far beyond the limit of the influence of dilution rate manipulation. Based on their technical parameters, we estimated dilution rates over the entire flow-cell channel from the reports of Heydorn et al. (2000) (*D* = 19 h^-1^), Foster and Kolenbrander (2004) (*D* = 50 h^-1^), and Nance et al. (2013) with Fernandez et al. (2017) combined (*D* between 65 and 260 h^-1^). While, due to (virtually laminar) plug flow in the flow cells, the actual dilution rates at any point would be even higher, the calculated values of *D* are far beyond the highest known growth rates for bacteria. Research of these systems underscores the understanding that in addition to the biological behavior of biofilms as a result of nutrient concentration and fluid flow, their physical properties determine their mechanical response to the flow (Melo and Vieira, 1999; Vinogradov et al., 2004). Shear stress has been commonly estimated in flow cell studies and is regarded as a key factor for the development of biofilms under these conditions (Fernandez et al., 2017). Biofilms were successfully maintained within the range of shear stress between 0.01 and 0.04 Pa (0.1–0.4 dyn cm^-2^; Fernandez et al., 2017) and particular features of biofilm architecture have been attributed to specific values of shear stress. The values between 0.8 and 2 Pa caused detachment of biofilms and were used to harvest the developed biofilms for genomic analysis (Benoit et al., 2010; Nance et al., 2013; Fernandez et al., 2017).

Even though a certain range of shear stress values has been found beneficial for building the biofilm architecture, a wider scale of shear stress spans from high values causing detachment, i.e., destruction of biofilms to low values, where no influence, detrimental, or beneficial could be observed. Both the *dilution rate* and the *shear stress* are apparently setting physical boundaries that limit the capacity of microorganisms for biofilm formation. Defining the domains within those boundaries may give us a predictive power for practical applications. Moreover, it could explain the evolution of colonizing wet surfaces formed on or within other (mostly multicellular) organisms including humans.

Although the two parameters are positively correlated with the rate of flow, their effective values depend on a set of additional conditions, such as a container shape and its dimensions, fluid viscosity, etc. Yet, we suggest that in the same hydraulic space, two separate boundary values of flow rate can be distinguished that control the biofilm development. Dilution rate appears to enhance the rate of biofilm formation up to a certain value, beyond which the acceleration of biofilm growth does not happen. Similarly, in the opposite way, the shear stress seems to restrict the biofilm formation down to a value, beyond which its influence is negligible.

Thus, for biofilm microorganisms, there may be three zones of flow involvement in their biofilm building capability:

(a) Slow flow domain of positive dilution rate influence that we addressed in this study, limited to dilution rates slower than 4 h^-1^.
(b) Intermediate flow domain where dilution rate or shear stress fluctuations have virtually no effect.
(c) Fast flow domain where dilution rates are substantially higher than 4 h^-1^ and shear stress alone selects the organisms capable of attachment to the substratum under the given conditions. Within this domain values around 0.01 Pa allow for biofilm buildup, whereas the shear stress 1 Pa and higher generally causes biofilm destruction.

The intermediate domain, by definition, represents the conditions where the biofilm buildup is maximized. Hence, those conditions should be met, where biofilms may be beneficial and avoided wherever biofilm formation (e.g., biofouling) is undesirable. The behavior and interactions of biofilm organisms reported in the flow cell studies are derived from the fast-flow domain, thus they do not necessarily characterize the same organisms growing under conditions of the slow-flow domain, and *vice versa*.

For the proposed boundary between the slow and intermediate domain, the implication of results of the present study is that the borderline value of flow/dilution rate is further controlled by the concentration of limiting nutrients.

## Conclusion

Experimental data describing the positive correlation between biofilm formation and dilution rate have been previously published referring to both clonal (Molin et al., 1982; Li et al., 2001) and mixed cultures of microorganisms: biofilm waste-water reactors (VanLoosdrecht and Heijnen, 1993); and defined mixture of species (Beyenal and Lewandowski, 2002). Yet, little consideration has been given thus far to the effect of suspended cell growth rate on nutrient concentration. Some methodology texts have avoided discussing involvement of flow except for its correlation with shear stress (Peterson et al., 2011). Occasionally, only flow rates instead of dilution rates have been made available (Pratten et al., 1998, 2000) which could lead to misinterpretation of results by other researchers in follow up studies. Conversely, clear understanding of the relationship between dilution rate and nutrient availability for the biofilms may simplify decision making in such diverse fields as managing water or sewage pipelines, designing bioreactors, and running biomedical laboratory experiments. Although more accurate description of the biofilm formation of *E. faecalis* can be achieved with a more data intensive study, our results demonstrated that in a defined range of the fluid exchange rate, the chemostat theory is a good predictor of the biofilm buildup.

## Author Contributions

ML contributed to the conception and study design; acquisition, analysis, data interpretation, and application of the mathematical model; and performed the experiments and wrote the manuscript. DC contributed to the study materials, analysis tools and reagents, and wrote the manuscript. DM contributed to the application of the mathematical model and wrote the manuscript.

## Conflict of Interest Statement

The authors declare that the research was conducted in the absence of any commercial or financial relationships that could be construed as a potential conflict of interest.
